# Supplementary material for: Association between statin use and clinical course, microbiologic characteristics, and long-term outcome of early Lyme borreliosis. A post hoc analysis of prospective clinical trials of adult patients with erythema migrans
Source: PLoS One. 2021 Dec 16;16(12):e0261194. doi: 10.1371/journal.pone.0261194 (PMC8675750; doi:10.1371/journal.pone.0261194)
Supplement: Attachment — Submitted filename: PlosOne 2021 Stupica etal.pdf [file pone.0261194.s001.pdf]

PONE

Review: Association between Statin Use and Clinical Course, Microbiologic Characteristics, and Long-Term Outcome or Early Lyme Borreliosis

This aim of this manuscript was to assess the impact of statins on the clinical course and outcome of patients with early Lyme borreliosis. The strengths of this study are a large patient cohort (n>1300) evaluated at a single medical center, and the systematic follow-up evaluation of these patients for 1 year. The manuscript is well written, and the conclusions are clear. I have only minor comments which are provided below.

1) In the Methods section (line 06-99), the authors make a distinction in defining “partial recovery” vs “treatment failure”, however no distinction is made between these groups in the manuscript. The rationale for this distinction is not clear. The groups could be combined into “incomplete recovery” in the Methods section, or additional explanation is needed for why the distinction is necessary. Did the authors find a difference in patients with incomplete recovery were stratified by “partial” vs “failure”?

2) Line 112-113. It would help to state that the second skin biopsy was taken 2-3 months after the start of antibiotic therapy.

3) Please clarify that Table 3 is the association of clinical characteristics at first visit, before antibiotics. This could go into the title for Table 3: Association between clinical characteristics at enrollment and incomplete recovery.

4) Line 195-197: This section is a better fit for the paragraph discussing the results in Table 2. It could follow the sentence on line 181.

5) Line 214-217: Although reference 12, which is based on mice, supports the upregulation of inflammatory responses to statins, references 25 and 26, which are based in humans, do not. Indeed, cytokine levels in patients with EM are generally higher at enrolment during acute infection than after antibiotic therapy / convalescence which goes along with the role of cytokines in the pathogenesis of this disease. One could argue that the greater inflammatory responses to statins could be advantageous and lead to Borrelia clearance, but they could also be disadvantageous by contributing to immune-associated symptoms. It is not clear how these points connect back with the idea that lipids may impact Borrelia immunogenicity. The paragraph should be rewritten and/or better references are needed in support of this point.
